# Chlorido(ethyl­diphenyl­phosphine-κ*P*)(1-pyrrolidinecarbodithio­ato-κ^2^ *S*,*S*′)nickel(II)

**DOI:** 10.1107/S1600536808011860

**Published:** 2008-04-30

**Authors:** Anna Kropidłowska, Ilona Turowska-Tyrk, Barbara Becker

**Affiliations:** a Department of Inorganic Chemistry, Chemical Faculty, Gdańsk University of Technology, 11/12 G. Narutowicza St., 80-952 PL Gdańsk, Poland; b Institute of Physical and Theoretical Chemistry, Chemical Faculty, Wrocław University of Technology, 27 Wybrzeże Wyspiańskiego, 50-370 PL Wrocław, Poland

## Abstract

In the crystal structure of the title complex, [Ni(C_5_H_8_NS_2_)Cl(C_14_H_15_P)], the Ni atom is coordinated by an *S*,*S*′-chelating dithio­carbamate, a chloride and a diphenyl­ethyl­phosphine ligand in a distorted square-planar arrangement.

## Related literature

For related literature, see: Allen (2002 ▶); Darkwa *et al.* (1999 ▶); Kropidłowska, Chojnacki *et al.* (2007 ▶); Kropidłowska, Janczak *et al.* (2007 ▶); Pastorek *et al.* (1996 ▶, 1999 ▶); Reger & Collins (1995 ▶).
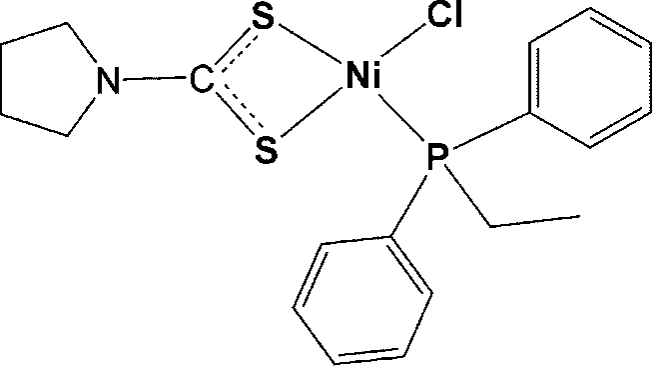


## Experimental

### 

#### Crystal data

[Ni(C_5_H_8_NS_2_)Cl(C_14_H_15_P)]
*M* *_r_* = 454.63
Monoclinic, 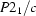

*a* = 6.5218 (5) Å
*b* = 19.1695 (15) Å
*c* = 16.6178 (14) Å
β = 90.786 (6)°
*V* = 2077.4 (3) Å^3^
*Z* = 4
Mo *K*α radiation
μ = 1.34 mm^−1^
*T* = 299 (2) K
0.50 × 0.21 × 0.17 mm

#### Data collection

Kuma KM-4-CCD diffractometer
Absorption correction: refined from Δ*F* (Walker & Stuart, 1983 ▶) *T*_min_ = 0.553, *T*_max_ = 0.804
10912 measured reflections
3637 independent reflections
2989 reflections with *I* > 2σ(*I*)
*R*_int_ = 0.042

#### Refinement

*R*[*F* ^2^ > 2σ(*F* ^2^)] = 0.040
*wR*(*F* ^2^) = 0.113
*S* = 1.08
3637 reflections
226 parameters
H-atom parameters constrained
Δρ_max_ = 0.57 e Å^−3^
Δρ_min_ = −0.35 e Å^−3^

### 

Data collection: *CrysAlis CCD* (Oxford Diffraction, 2003 ▶); cell refinement: *CrysAlis CCD*; data reduction: *CrysAlis RED* (Oxford Diffraction, 2003 ▶); program(s) used to solve structure: *SHELXS97* (Sheldrick, 2008 ▶); program(s) used to refine structure: *SHELXL97* (Sheldrick, 2008 ▶); molecular graphics: *ORTEP-3* (Farrugia, 1997 ▶) and *Mercury* (Macrae *et al.*, 2006 ▶); software used to prepare material for publication: *SHELXL97*.

## Supplementary Material

Crystal structure: contains datablocks global, I. DOI: 10.1107/S1600536808011860/at2563sup1.cif

Structure factors: contains datablocks I. DOI: 10.1107/S1600536808011860/at2563Isup2.hkl

Additional supplementary materials:  crystallographic information; 3D view; checkCIF report

## Figures and Tables

**Fig. 1.**
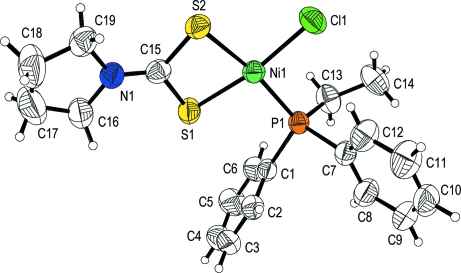
Molecular structure and atom-numbering scheme for the title complex (I) with displacement ellipsoids drawn at 50% probability level. H atoms are represented as circles of arbitrary size.

**Fig. 2.**
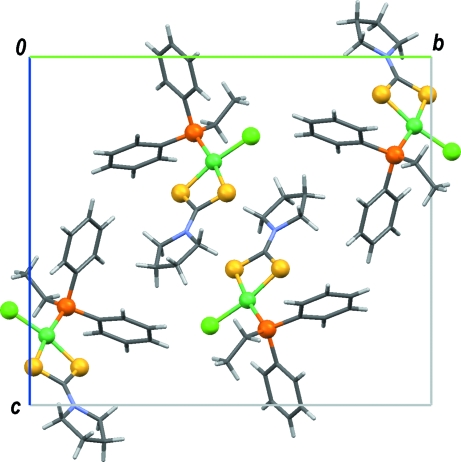
Schematic drawing of the crystal packing down the a axis.

## supplementary crystallographic information

### Comment

Metal (Ni, Pd) complexes in which the atom is coordinated by a *S,S-*chelating dithiocarbamate, one halogenide and one phosphine have been investigated and used to obtain compounds with a sulfur rich kernel arising from the presence of two different *S-*donor ligands (Darkwa *et al.*, 1999; Pastorek *et al.*, 1999; Reger & Collins, 1995). Several structures of such species are stored in the Cambridge Structural Database (CSD-2007, Allen 2002).

Recently, we reported the synthesis of [Ni{S_2_CN(CH_2_)_4_}(Cl)(PPh_3_)] (Kropidłowska, Janczak *et al.*, 2007) solvated by a chloroform molecule, which interacts with the complex by a weak C—H···S hydrogen bond. The structure of homologous hemisolvated [Ni{S_2_CN(CH_2_)_4_}(Br)(PPh_3_)] has also been reported (Pastorek *et al.*, 1996). In the present paper we describe the structure of another nickel(II) complex - (1-pyrrolidinylcarbodithioato-S,*S*') -chlorido-(diphenylethylphosphine)nickel(II), [Ni{S_2_CN(CH_2_)_4_}(Cl)(PPh_2_Et)] (I) obtained by essentially quantitative metathesis of *trans*-dichloro-bis(diphenylethylphosphine)-nickel(II) and bis(1-pyrrolidinylcarbodithioato-S,*S*')nickel(II). The molecular structure of (I) with the atom numbering scheme is shown in Figure 1.

In this compound the metal(II) ion is four-coordinated within a typical square planar [NiClS_2_P] heterogeneous coordination sphere. The dithiocarbamate ligand acts as a bidentate chelate, coordinating to Ni *via* both S atoms and thus introducing a deformation of the coordination geometry. Atom S1 is located *trans *to the Cl ligand and atom S2 is *trans* to the diphenylethylphosphine ligand. Although (I) was obtained in the same manner as previously mentioned [Ni{S_2_CN(CH_2_)_4_}(Cl)(PPh_3_)] it did not retain the solvent within its crystal structure, similarily to previously described [Ni{S_2_CN(C_4_H_8_O)}(Cl)(PPh_3_)] (Kropidłowska, Chojnacki *et al.*, 2007). The schematic drawing of the crystal packing in (I) is presented in Figure 2.

### Experimental

Nickel chloride, NiCl_2_^.^6H_2_O (0.594 g, 0.0025 mol, purchased from POCh) was dissolved in 50 ml of methanol/water (10/1, *v*/*v*) and this solution was added dropwise to the ammonium salt of pyrrolidinylcarbodithioic acid C_4_H_8_NCS_2_NH_4_ (0.82 g, 0.005 mol, Fluka) dissolved in methanol/water. This mixture was stirred vigorously under argon atmosphere for *ca* 20 minutes, then filtered and the filtrate left for crystallization at 278 K. After a week the green crystalline product, namely Ni(S_2_CNC_4_H_8_)_2_ was collected. It was further dissolved (0.199 g, 0.00057 mol) in 10 ml of chloroform and mixed with solution of equimolar amount of NiCl_2_(PPh_2_Et)_2_ (0.315 g). The mixture which turned into deep violet colour, was stirred for 10 minutes and then filtered. To the filtrate 10 ml of Et_2_O was added. After two days violet crystals were collected and washed with several portions of ether.

### Refinement

All H atoms were positioned geometrically and treated as riding with C—H = 0.93 - 0.97 Å, and with *U*_iso_(H) values of 1.2×*U*_eq_ of the parent methylene carbon and *U*_iso_(H) values of 1.5x*U*_eq_ of the methyl group carbon.

### Crystal data

**Table:**

[Ni(C_5_H_8_NS_2_)Cl(C_14_H_15_P)]	*F*_000_ = 944
*M**_r_* = 454.63	*D*_x_ = 1.454 Mg m^−^^3^
Monoclinic, *P*2_1_/*c*	Mo *K*α radiation λ = 0.71073 Å
Hall symbol: -P 2ybc	Cell parameters from 3645 reflections
*a* = 6.5218 (5) Å	θ = 3.5–23.0º
*b* = 19.1695 (15) Å	µ = 1.34 mm^−^^1^
*c* = 16.6178 (14) Å	*T* = 299 (2) K
β = 90.786 (6)º	Block, violet
*V* = 2077.4 (3) Å^3^	0.50 × 0.21 × 0.17 mm
*Z* = 4	

### Data collection

**Table:**

Kuma KM-4-CCD diffractometer	3637 independent reflections
Radiation source: fine-focus sealed tube	2989 reflections with *I* > 2σ(*I*)
Monochromator: graphite	*R*_int_ = 0.042
*T* = 299(2) K	θ_max_ = 25.0º
ω scans	θ_min_ = 3.1º
Absorption correction: part of the refinement model (Δ*F*)(Walker & Stuart, 1983)	*h* = −6→7
*T*_min_ = 0.553, *T*_max_ = 0.804	*k* = −22→22
10912 measured reflections	*l* = −19→18

### Refinement

**Table:**

Refinement on *F*^2^	Secondary atom site location: difference Fourier map
Least-squares matrix: full	Hydrogen site location: inferred from neighbouring sites
*R*[*F*^2^ > 2σ(*F*^2^)] = 0.040	H-atom parameters constrained
*wR*(*F*^2^) = 0.113	*w* = 1/[σ^2^(*F*_o_^2^) + (0.0634*P*)^2^ + 0.6647*P*] where *P* = (*F*_o_^2^ + 2*F*_c_^2^)/3
*S* = 1.08	(Δ/σ)_max_ = 0.001
3637 reflections	Δρ_max_ = 0.57 e Å^−^^3^
226 parameters	Δρ_min_ = −0.35 e Å^−^^3^
Primary atom site location: structure-invariant direct methods	Extinction correction: none

### Special details

**Table:**

Geometry. All e.s.d.'s (except the e.s.d. in the dihedral angle between two l.s. planes) are estimated using the full covariance matrix. The cell e.s.d.'s are taken into account individually in the estimation of e.s.d.'s in distances, angles and torsion angles; correlations between e.s.d.'s in cell parameters are only used when they are defined by crystal symmetry. An approximate (isotropic) treatment of cell e.s.d.'s is used for estimating e.s.d.'s involving l.s. planes.
Refinement. Refinement of *F*^2^ against ALL reflections. The weighted *R*-factor *wR* and goodness of fit *S* are based on *F*^2^, conventional *R*-factors *R* are based on *F*, with *F* set to zero for negative *F*^2^. The threshold expression of *F*^2^ > σ(*F*^2^) is used only for calculating *R*-factors(gt) *etc*. and is not relevant to the choice of reflections for refinement. *R*-factors based on *F*^2^ are statistically about twice as large as those based on *F*, and *R*- factors based on ALL data will be even larger.

### Fractional atomic coordinates and isotropic or equivalent isotropic displacement parameters (Å^2^)

**Table:**

	*x*	*y*	*z*	*U*_iso_*/*U*_eq_	
Ni1	0.41398 (6)	0.542285 (19)	0.69879 (2)	0.04145 (15)	
Cl1	0.45930 (14)	0.44894 (4)	0.77213 (6)	0.0630 (3)	
P1	0.19702 (11)	0.59106 (4)	0.78208 (5)	0.0397 (2)	
S1	0.38209 (13)	0.62417 (5)	0.60835 (5)	0.0544 (2)	
S2	0.64891 (13)	0.50962 (4)	0.60948 (5)	0.0508 (2)	
N1	0.6761 (4)	0.61076 (14)	0.49827 (16)	0.0524 (7)	
C1	0.0929 (4)	0.67350 (15)	0.74538 (17)	0.0421 (7)	
C2	0.2112 (5)	0.73347 (17)	0.7499 (2)	0.0534 (8)	
H2	0.3426	0.7314	0.7722	0.064*	
C3	0.1353 (7)	0.79645 (19)	0.7214 (2)	0.0658 (10)	
H3	0.2148	0.8366	0.7255	0.079*	
C4	−0.0576 (7)	0.7995 (2)	0.6872 (3)	0.0732 (11)	
H4	−0.1092	0.8418	0.6686	0.088*	
C5	−0.1733 (7)	0.7405 (2)	0.6805 (3)	0.0743 (11)	
H5	−0.3029	0.7426	0.6566	0.089*	
C6	−0.0994 (5)	0.67784 (19)	0.7092 (2)	0.0571 (9)	
H6	−0.1796	0.6379	0.7041	0.069*	
C7	0.2986 (5)	0.61533 (16)	0.88112 (18)	0.0447 (7)	
C8	0.1922 (6)	0.6611 (2)	0.9294 (2)	0.0651 (10)	
H8	0.0725	0.6817	0.9097	0.078*	
C9	0.2601 (8)	0.6766 (2)	1.0060 (2)	0.0790 (12)	
H9	0.1865	0.7076	1.0376	0.095*	
C10	0.4359 (8)	0.6466 (2)	1.0358 (2)	0.0769 (12)	
H10	0.4819	0.6569	1.0876	0.092*	
C11	0.5419 (7)	0.6020 (3)	0.9890 (3)	0.0833 (13)	
H11	0.6618	0.5818	1.0090	0.100*	
C12	0.4748 (5)	0.5858 (2)	0.9115 (2)	0.0637 (10)	
H12	0.5495	0.5549	0.8802	0.076*	
C13	−0.0307 (5)	0.53894 (19)	0.8047 (2)	0.0614 (9)	
H13A	−0.1441	0.5706	0.8141	0.074*	
H13B	−0.0657	0.5114	0.7575	0.074*	
C14	−0.0117 (6)	0.4907 (2)	0.8755 (3)	0.0737 (11)	
H14A	−0.1381	0.4659	0.8821	0.111*	
H14B	0.0179	0.5174	0.9232	0.111*	
H14C	0.0973	0.4581	0.8666	0.111*	
C15	0.5834 (5)	0.58493 (17)	0.55992 (19)	0.0468 (7)	
C16	0.6134 (7)	0.6751 (2)	0.4568 (2)	0.0740 (11)	
H16A	0.4787	0.6699	0.4321	0.089*	
H16B	0.6106	0.7141	0.4940	0.089*	
C17	0.7689 (10)	0.6853 (3)	0.3962 (4)	0.125 (2)	
H17A	0.7055	0.6832	0.3431	0.151*	
H17B	0.8298	0.7312	0.4028	0.151*	
C18	0.9245 (7)	0.6337 (3)	0.4024 (3)	0.1021 (17)	
H18A	1.0527	0.6550	0.4198	0.123*	
H18B	0.9453	0.6122	0.3503	0.123*	
C19	0.8606 (5)	0.5801 (2)	0.4617 (2)	0.0609 (9)	
H19A	0.8285	0.5362	0.4353	0.073*	
H19B	0.9673	0.5722	0.5020	0.073*	

### Atomic displacement parameters (Å^2^)

**Table:**

	*U*^11^	*U*^22^	*U*^33^	*U*^12^	*U*^13^	*U*^23^
Ni1	0.0434 (2)	0.0415 (2)	0.0394 (2)	0.00158 (16)	−0.00237 (16)	−0.00039 (16)
Cl1	0.0697 (6)	0.0521 (5)	0.0672 (6)	0.0089 (4)	0.0010 (4)	0.0144 (4)
P1	0.0380 (4)	0.0437 (4)	0.0373 (4)	−0.0021 (3)	−0.0015 (3)	0.0024 (3)
S1	0.0597 (5)	0.0577 (5)	0.0459 (5)	0.0158 (4)	0.0092 (4)	0.0086 (4)
S2	0.0543 (5)	0.0506 (5)	0.0475 (5)	0.0093 (4)	0.0022 (4)	−0.0024 (4)
N1	0.0547 (16)	0.0542 (16)	0.0486 (15)	0.0051 (13)	0.0081 (13)	0.0023 (13)
C1	0.0450 (16)	0.0463 (17)	0.0351 (15)	0.0027 (13)	0.0054 (12)	0.0009 (13)
C2	0.0593 (19)	0.0530 (19)	0.0481 (18)	−0.0062 (16)	0.0068 (15)	0.0000 (15)
C3	0.085 (3)	0.050 (2)	0.063 (2)	−0.0068 (19)	0.019 (2)	0.0035 (17)
C4	0.091 (3)	0.054 (2)	0.074 (3)	0.022 (2)	0.010 (2)	0.0109 (19)
C5	0.073 (2)	0.067 (3)	0.082 (3)	0.018 (2)	−0.008 (2)	0.013 (2)
C6	0.056 (2)	0.057 (2)	0.058 (2)	0.0034 (16)	−0.0081 (16)	0.0028 (16)
C7	0.0473 (17)	0.0488 (17)	0.0381 (16)	−0.0055 (13)	−0.0004 (13)	0.0008 (13)
C8	0.077 (2)	0.070 (2)	0.048 (2)	0.0109 (19)	−0.0018 (17)	−0.0049 (18)
C9	0.116 (4)	0.074 (3)	0.047 (2)	0.000 (3)	0.008 (2)	−0.008 (2)
C10	0.105 (3)	0.078 (3)	0.048 (2)	−0.025 (3)	−0.014 (2)	0.001 (2)
C11	0.074 (3)	0.111 (4)	0.064 (3)	0.001 (2)	−0.029 (2)	−0.002 (3)
C12	0.0516 (19)	0.086 (3)	0.054 (2)	0.0034 (18)	−0.0077 (16)	−0.0089 (19)
C13	0.0479 (19)	0.070 (2)	0.066 (2)	−0.0158 (16)	−0.0042 (16)	0.0143 (18)
C14	0.061 (2)	0.066 (2)	0.094 (3)	−0.0119 (18)	0.002 (2)	0.027 (2)
C15	0.0475 (17)	0.0486 (18)	0.0443 (17)	0.0054 (14)	−0.0042 (13)	−0.0050 (14)
C16	0.090 (3)	0.069 (2)	0.064 (2)	0.011 (2)	0.020 (2)	0.015 (2)
C17	0.145 (5)	0.091 (4)	0.142 (5)	0.014 (4)	0.076 (4)	0.045 (4)
C18	0.081 (3)	0.127 (4)	0.100 (4)	0.016 (3)	0.043 (3)	0.042 (3)
C19	0.0510 (19)	0.070 (2)	0.062 (2)	−0.0008 (17)	0.0124 (16)	−0.0050 (18)

### Geometric parameters (Å, °)

**Table:**

Ni1—S1	2.1812 (9)	C8—H8	0.9300
Ni1—Cl1	2.1828 (9)	C9—C10	1.369 (6)
Ni1—P1	2.2014 (8)	C9—H9	0.9300
Ni1—S2	2.2371 (9)	C10—C11	1.353 (6)
P1—C1	1.822 (3)	C10—H10	0.9300
P1—C7	1.826 (3)	C11—C12	1.389 (5)
P1—C13	1.833 (3)	C11—H11	0.9300
S1—C15	1.722 (3)	C12—H12	0.9300
S2—C15	1.713 (3)	C13—C14	1.501 (5)
N1—C15	1.295 (4)	C13—H13A	0.9700
N1—C16	1.468 (5)	C13—H13B	0.9700
N1—C19	1.477 (4)	C14—H14A	0.9600
C1—C2	1.386 (4)	C14—H14B	0.9600
C1—C6	1.386 (4)	C14—H14C	0.9600
C2—C3	1.385 (5)	C16—C17	1.453 (6)
C2—H2	0.9300	C16—H16A	0.9700
C3—C4	1.375 (6)	C16—H16B	0.9700
C3—H3	0.9300	C17—C18	1.420 (7)
C4—C5	1.362 (6)	C17—H17A	0.9700
C4—H4	0.9300	C17—H17B	0.9700
C5—C6	1.377 (5)	C18—C19	1.488 (5)
C5—H5	0.9300	C18—H18A	0.9700
C6—H6	0.9300	C18—H18B	0.9700
C7—C12	1.371 (5)	C19—H19A	0.9700
C7—C8	1.382 (5)	C19—H19B	0.9700
C8—C9	1.375 (5)		
			
S1—Ni1—Cl1	170.25 (4)	C9—C10—H10	120.3
S1—Ni1—P1	94.10 (3)	C10—C11—C12	121.1 (4)
Cl1—Ni1—P1	94.62 (3)	C10—C11—H11	119.5
S1—Ni1—S2	78.70 (3)	C12—C11—H11	119.5
Cl1—Ni1—S2	92.99 (4)	C7—C12—C11	120.1 (4)
P1—Ni1—S2	171.11 (4)	C7—C12—H12	120.0
C1—P1—C7	102.11 (14)	C11—C12—H12	120.0
C1—P1—C13	104.01 (16)	C14—C13—P1	116.0 (3)
C7—P1—C13	103.83 (16)	C14—C13—H13A	108.3
C1—P1—Ni1	113.44 (9)	P1—C13—H13A	108.3
C7—P1—Ni1	116.52 (10)	C14—C13—H13B	108.3
C13—P1—Ni1	115.28 (14)	P1—C13—H13B	108.3
C15—S1—Ni1	86.65 (11)	H13A—C13—H13B	107.4
C15—S2—Ni1	85.09 (11)	C13—C14—H14A	109.5
C15—N1—C16	124.2 (3)	C13—C14—H14B	109.5
C15—N1—C19	124.4 (3)	H14A—C14—H14B	109.5
C16—N1—C19	111.4 (3)	C13—C14—H14C	109.5
C2—C1—C6	118.3 (3)	H14A—C14—H14C	109.5
C2—C1—P1	119.8 (2)	H14B—C14—H14C	109.5
C6—C1—P1	121.9 (2)	N1—C15—S2	125.9 (2)
C3—C2—C1	120.6 (3)	N1—C15—S1	124.7 (2)
C3—C2—H2	119.7	S2—C15—S1	109.28 (18)
C1—C2—H2	119.7	C17—C16—N1	104.2 (3)
C4—C3—C2	119.9 (4)	C17—C16—H16A	110.9
C4—C3—H3	120.1	N1—C16—H16A	110.9
C2—C3—H3	120.1	C17—C16—H16B	110.9
C5—C4—C3	120.1 (4)	N1—C16—H16B	110.9
C5—C4—H4	119.9	H16A—C16—H16B	108.9
C3—C4—H4	119.9	C18—C17—C16	111.2 (4)
C4—C5—C6	120.3 (4)	C18—C17—H17A	109.4
C4—C5—H5	119.8	C16—C17—H17A	109.4
C6—C5—H5	119.8	C18—C17—H17B	109.4
C5—C6—C1	120.8 (4)	C16—C17—H17B	109.4
C5—C6—H6	119.6	H17A—C17—H17B	108.0
C1—C6—H6	119.6	C17—C18—C19	108.8 (4)
C12—C7—C8	118.2 (3)	C17—C18—H18A	109.9
C12—C7—P1	121.3 (3)	C19—C18—H18A	109.9
C8—C7—P1	120.4 (3)	C17—C18—H18B	109.9
C9—C8—C7	121.1 (4)	C19—C18—H18B	109.9
C9—C8—H8	119.4	H18A—C18—H18B	108.3
C7—C8—H8	119.4	N1—C19—C18	103.6 (3)
C10—C9—C8	120.1 (4)	N1—C19—H19A	111.0
C10—C9—H9	120.0	C18—C19—H19A	111.0
C8—C9—H9	120.0	N1—C19—H19B	111.0
C11—C10—C9	119.4 (4)	C18—C19—H19B	111.0
C11—C10—H10	120.3	H19A—C19—H19B	109.0
